# Supplementary material for: What dictates soft clay-like lithium superionic conductor formation from rigid salts mixture
Source: Nat Commun. 2023 Oct 28;14:6884. doi: 10.1038/s41467-023-42538-2 (PMC10613223; doi:10.1038/s41467-023-42538-2)
Supplement: Supplementary file 1 — Supplementary Information [file 41467_2023_42538_MOESM1_ESM.pdf]

Supplementary Information

What dictates soft clay-like Lithium superionic conductor formation from rigid-salts mixture

Sunny Gupta et. al

Supplementary Note 1: Training, validating, and benchmarking DeepMD PE model

Supplementary Note 1.1: Training the DeepMD PE model

To train the DeepMD PE model, atomic configurations of 13 stable crystalline phases (unary, binary, and ternary), marked red, in the Ga-F-Li-Cl quaternary chemical space (Supplementary Figure 1 a-d) along with slab-like structures of LiCl|GaF_3 with different thicknesses (Supplementary Figure 1 e-g) were used. The slab-like structures were constructed from multilayer slabs of [001] LiCl and [001] GaF_3, which were vertically interfaced "|", and separated by 3 Å. A maximum strain of ~5 % was applied to match the lattice constants of LiCl and GaF_3 slabs in the LiCl|GaF_3 geometry. The training data consisted of atomic configurations obtained by melting and quenching the different systems (specified in Supplementary Figure 1 and Supplementary Table 1) using AIMD simulations with parameters listed (see Notes) in Supplementary Table 1. In total, over 700k AIMD frames were generated, out of which 250k atomic configurations were randomly selected to train the DeepMD model. Among the 250k configurations, 80% of them were used for training and 20% for validation. The DeepMD model was trained for $8x10^5$ iterations until the root mean square error (RMSE) in energy and forces became constant, with the RMSE values for energy and forces being <1 meV/atom, and <70 meV/Å, respectively (see Supplementary Figure 2).

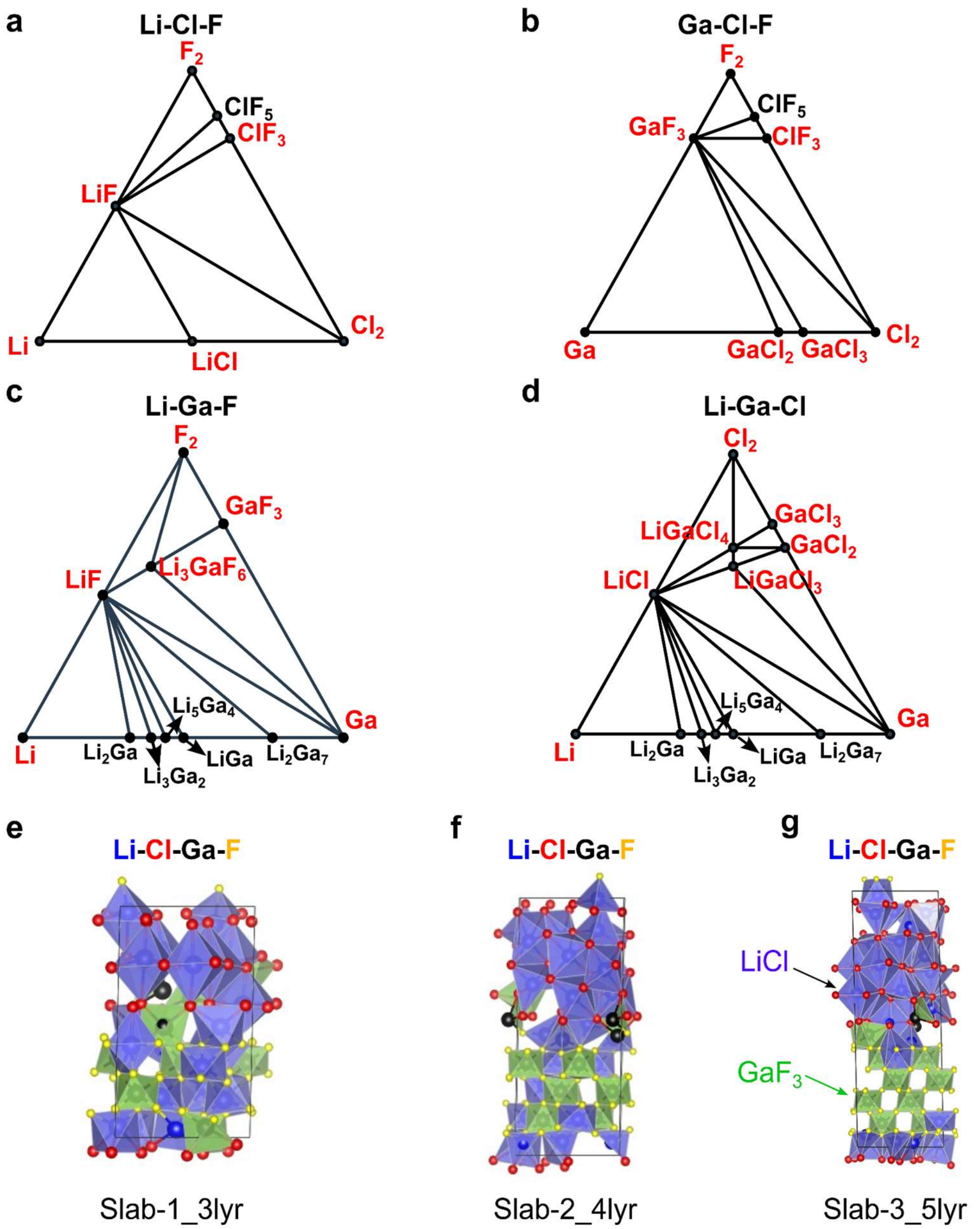

Supplementary Figure 1: Chemical systems used for training the interatomic potential energy model for the Ga-F-Li-Cl chemical space. Ternary phase diagrams of (a) Li-Cl-F, (b) Ga-Cl-F, (c) Li-Ga-F, and (d) Li-Ga-Cl. Only systems marked in red were used for training. (e, f, g) Slab-like structures of LiCl|GaF_3, where multilayer slabs of [001] LiCl and [001] GaF_3 are interfaced "|" in a vertical geometry and have different thicknesses.

Supplementary Table 1: Different chemical systems used in the training and validation of DeePMD PE model

system	#atoms	#frames	probability	Notes
$GaCl_3$	64	10000	0.03	NVT - Melt and quench, T = 0 - 400 K, t = 50 ps
$GaCl_3$-NPT	64	10000	0.02	NPT - T = 400 K, t = 50 ps
GaF_3	216	7500	0.04	NVT - Melt and quench, T = 0 - 1100 K, t = 50 ps
GaF_3-NPT	216	5000	0.03	NPT - T = 1100 K, t = 50 ps
$GaCl_2$	192	7500	0.02	NVT - Melt and quench, T = 0 - 400 K, t = 50 ps
$GaCl_2$-NPT	192	5000	0.02	NPT - T = 400 K, t = 50 ps
LiF	128	15000	0.015	NVT - Melt and quench, T = 0 - 1200 K, t = 50 ps
LiF-NPT	128	15000	0.015	NPT - T = 1200 K, t = 50 ps
LiCl	128	15000	0.02	NVT - Melt and quench, T = 0 - 1000 K, t = 50 ps
LiCl-NPT	128	15000	0.02	NPT - T = 1000 K, t = 50 ps
F_2	64	10000	0.02	NVT - Melt and quench, T = 0 - 400 K, t = 50 ps
ClF_3	128	10000	0.02	NVT - Melt and quench, T = 0 - 400 K, t = 50 ps
Li-bulk	108	10000	0.02	NVT - Melt and quench, T = 0 - 500 K, t = 50 ps
Ga-bulk	144	10000	0.02	NVT - Melt and quench, T = 0 - 400 K, t = 50 ps
Cl_2	64	10000	0.02	NVT - Melt and quench, T = 0 - 400 K, t = 50 ps
$LiGaCl_4$	96	10000	0.02	NVT - Melt and quench, T = 0 - 550 K, t = 50 ps
$LiGaCl_3$	160	7500	0.02	NVT - Melt and quench, T = 0 - 550 K, t = 50 ps
Li_3GaF_6	120	7500	0.02	NVT - Melt and quench, T = 0 - 900 K, t = 50 ps
Li-Cl-Ga-F_3lyr ± 5% volumetric strained structures	96	15000	0.1	NVT - Melt and quench, T = 0 - 1100 K, t = 50 ps
Li-Cl-Ga-F_3lyr	96	15000	0.1	NPT – T = 1100 K, t = 50 ps
Li-Cl-Ga-F_4lyr ± 5% volumetric strained structures	128	15000	0.1	NVT - Melt and quench, T = 0 - 1100 K, t = 50 ps
Li-Cl-Ga-F_5lyr	160	10000	0.18	NVT - Melt and quench, T = 0 - 1100 K, t = 50 ps
Li-Cl-Ga-F_5lyr	160	15000	0.13	NPT – T = 1100 K, t = 50 ps

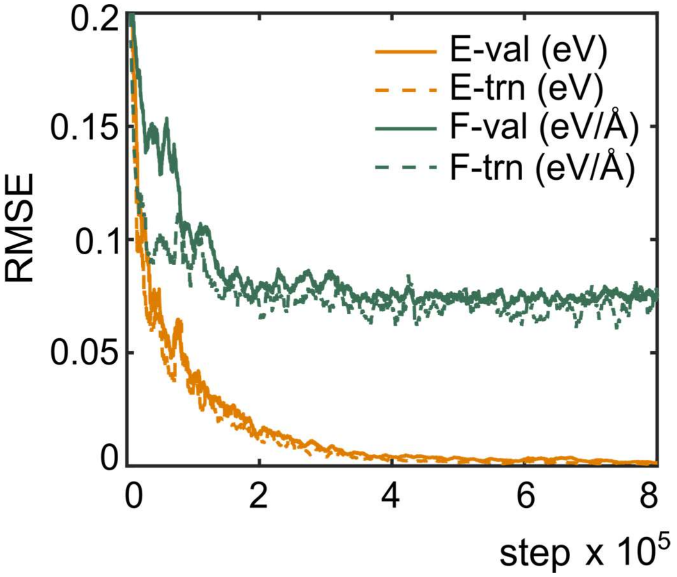

Supplementary Figure 2. The evolution of root-mean-squared-error (RMSE) in forces (*F*) and energy/atom (*E*) during the deep neural network training (trn) and validation (val) steps.

Supplementary Note 1.2: Testing and benchmarking trained DeepMD PE model

To assess the accuracy of the trained DeepMD PE model, several atomic configurations not included in the training and validation set were used for testing. Supplementary Figure 3 displays some examples of model testing, where the test structures were derived from slab-like geometry of LiCl|GaF_3 with varied thicknesses (Supplementary Figure 3 a,b) and a random Li-Cl-Ga-F structure constructed from only the LiCl and GaF_3 monomer molecules (initial structure in Supplementary Figure 3c). The slab-like structures were constructed from multilayer slabs of [001] LiCl and [001] GaF_3, which were vertically interfaced "|", and separated by 3 Å. A maximum strain of ~5 % was applied to match the lattice constants of LiCl and GaF_3 slabs in the LiCl|GaF_3 geometry. AIMD simulations of these test structures were performed at different temperatures (see caption Supplementary Figure 3) to generate the atomic configurations, and the trained DeepMD PE model was tested on these configurations. The comparison of energy and forces predicted by the DeepMD PE model and actual AIMD-DFT calculations is shown in the bottom panels of Supplementary Figure 3. The small RMSE error in both energy and forces indicates the high accuracy of our model.

The accuracy of the trained DeepMD PE model in analyzing the dynamics of Ga-F-Li-Cl chemical space was further evaluated through benchmarking against DFT-AIMD calculations of tensile stress-strain response and Li-ion conductivity in test structures. A comparison of the tensile stress-strain response of a test structure obtained from DFT-AIMD simulations and classical MD simulations using LAMMPS with the DeepMD PE model is presented in Supplementary Figure 4, which demonstrates a good agreement. Furthermore, Supplementary Table 2 displays a comparison of the bulk modulus and Li-ion conductivity obtained through the DeepMD-LAMMPS and DFT-AIMD calculations, which again show good agreement. These findings confirm the accuracy of the trained DeepMD model for studying the dynamics of Ga-F-Li-Cl chemical space.

To obtain the tensile stress-strain response, presented in Supplementary Figure 4b, of the

test structure shown in Supplementary Figure 4a, MD simulations were carried out at T = 300 K using both DFT-AIMD and DeepMD-LAMMPS. The simulations were conducted under the NVT ensemble, with a starting tensile strain of 0.01 applied along the z-direction. The simulation was done for t = 3 ps at the fixed strained volume, and the average stress σ_z was calculated. The structure obtained after this simulation was used as the initial structure for the next calculation, and a total tensile strain of 0.02 was applied along the z-direction. The simulation was again done for t = 3 ps at the fixed strained volume, and the average stress σ_z was evaluated. This procedure was repeated until the total tensile strain along the z-direction reached 0.1. Additionally, the Li-ion conductivity was computed from the mean squared displacements of Li-ions during the MD simulations and using the Nernst–Einstein approximation, which is based on the methodology established in a previous work[1]. MD simulations were carried out under the NVT ensemble at the respective temperatures shown in Supplementary Table 2 using both AIMD-DFT and DeepMD-LAMMPS.

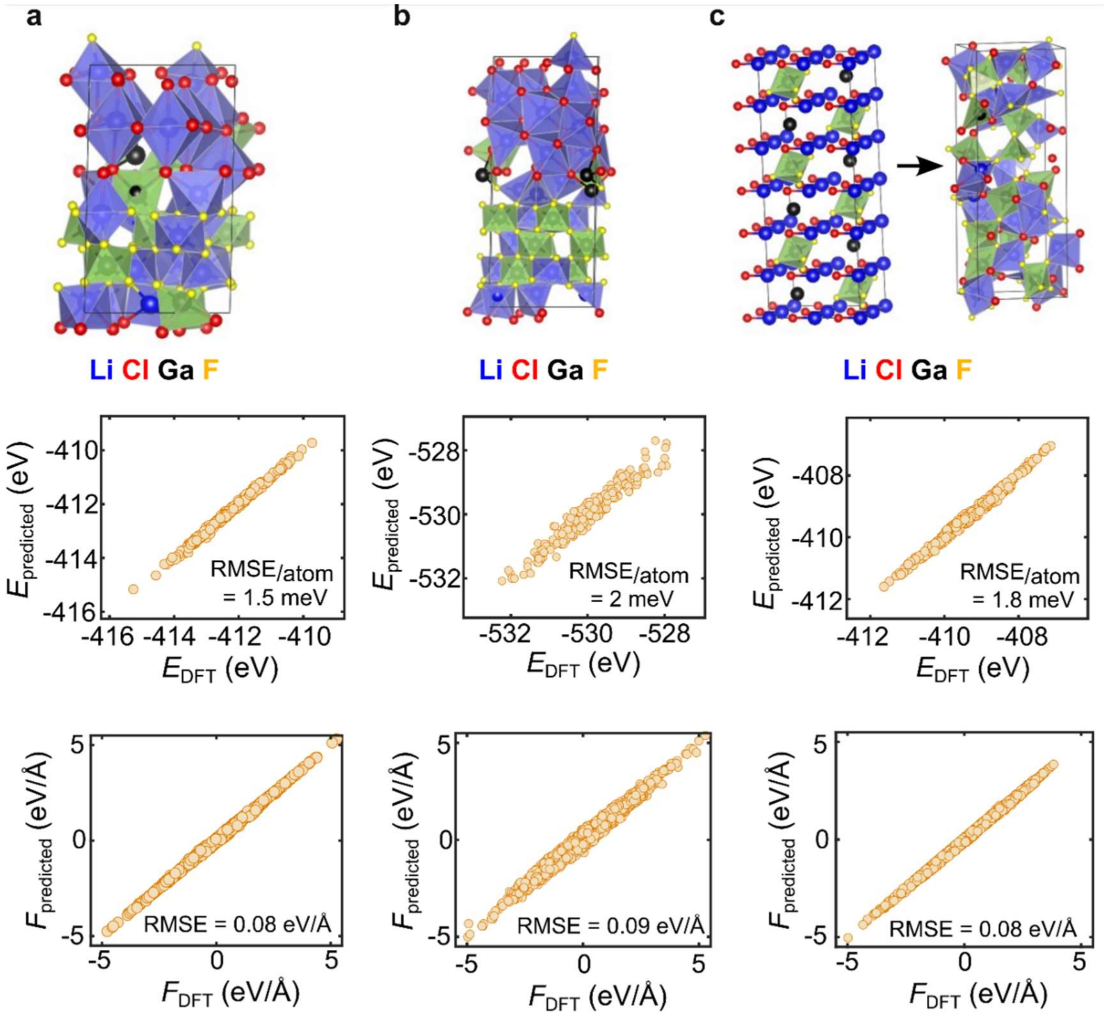

Supplementary Figure 3: Comparison of energy and forces computed by trained DeepMD PE model and DFT-AIMD for various structures (a) NVT AIMD run at T = 800 K of a Ga-F-Li-Cl structure with a slab like geometry, (b) NVT AIMD run at T = 750 K of another Ga-F-Li-Cl structure with a slab like geometry, and (c) NVT AIMD run at T = 800 K of a random Ga-F-Li-Cl structure constructed from LiCl and GaF_3 monomers.

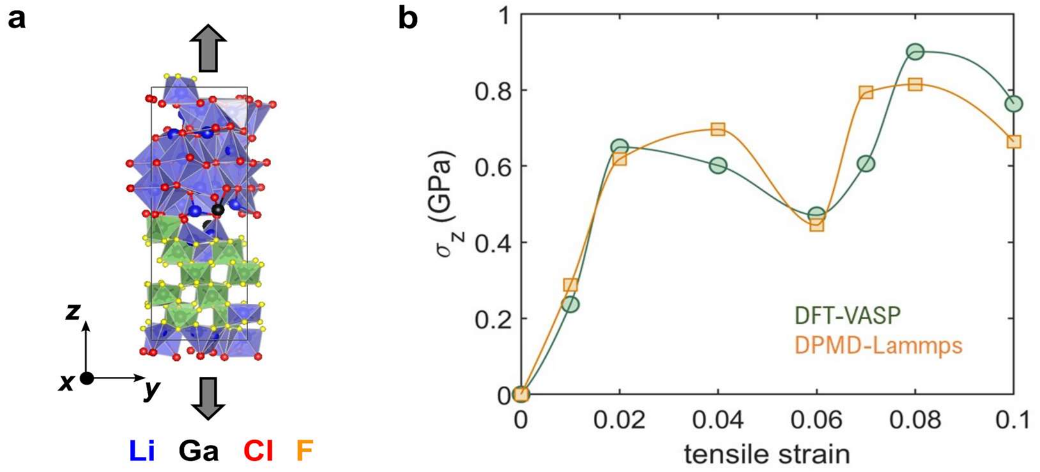

Supplementary Figure 4: Comparison of tensile stress-strain response at *T* = 300 K of a Ga-F-Li-Cl structure shown in (a) and its stress (σ_z) - strain response shown in (b). In (b) the values calculated with DFT-AIMD and DeePMD-LAMMPS are compared.

Supplementary Table 2: A comparison of properties of structure in Supplementary Figure 4 by trained DeePMD PE model and DFT calculations

Property	DFT (VASP)	DeePMD (LAMMPS)
Bulk Modulus	34 GPa	36 GPa
Li-ion conductivity T = 750 K	154 mS/cm	159 mS/cm
Li-ion conductivity T = 800 K	215 mS/cm	223 mS/cm
Li-ion conductivity T = 850 K	311 mS/cm	315 mS/cm

Supplementary Note 1.3: Testing the accuracy of DeepMD PE model on properties of amorphous systems

The trained DeepMD PE model was further tested on the properties of different amorphous systems in the Ga-F-Li-Cl chemical space. Amorphous phases of different chemical systems LiCl, LiF, $GaCl_3$, $LiGaCl_4$, and Li_3GaF_6 were created by melting and quenching each of the crystalline phases using AIMD-DFT and DeepMD-LAMMPS (DP) model. Melting was obtained by performing NVT simulation for each of the structures at T = 3000 K for t = 5 ps. The atomic configuration at t = 5 ps was subsequently relaxed at T = 0 K to obtain the amorphous phase. Supplementary Figure 5 shows a comparison of the radial pair distribution function of the amorphous systems, the energy of the amorphous systems with respect to their crystalline phase, and the density of the amorphous systems obtained with DFT and the trained DP model. The results obtained with DFT and the trained DP model are in good agreement.

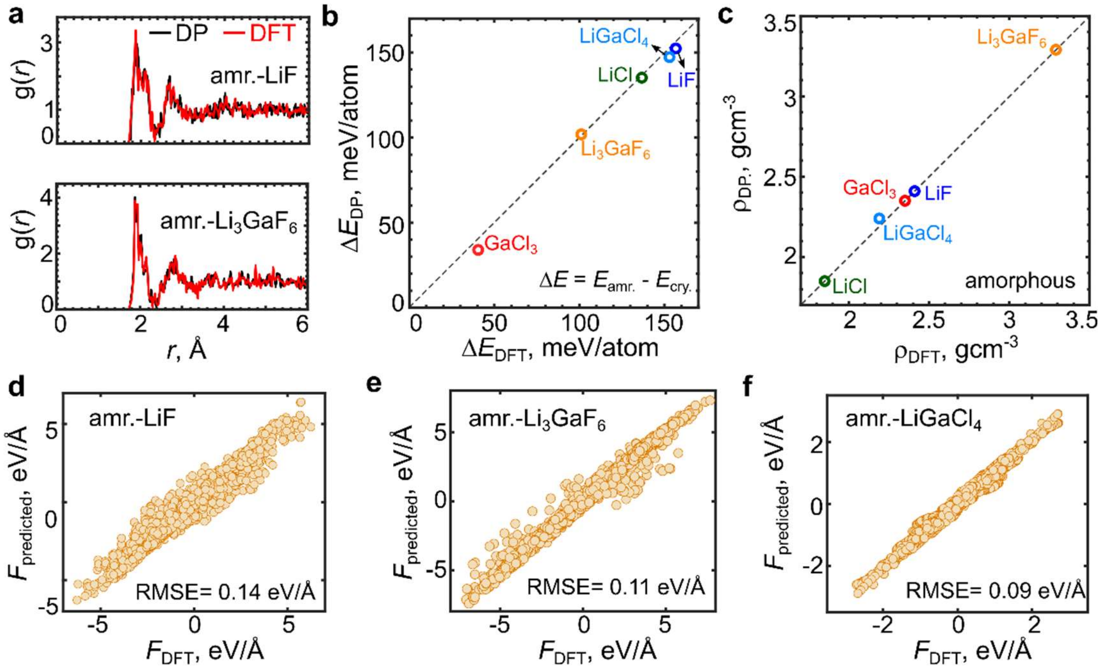

Supplementary Figure 5: (a) Comparison of the radial pair distribution function g(r) of amorphous (amr.) LiF and amorphous Li_3GaF_6 obtained with AIMD-DFT and trained DP model. (b) The relative energy of the amorphous (amr.) phase of different chemical systems with respect to their crystalline (cry.) phase obtained with AIMD-DFT and trained DP model. (c) Comparison of the density of different amorphous systems obtained with AIMD-DFT and the trained DP model. (d - f) Parity plot showing the atomic forces obtained by AIMD-DFT simulations at T = 3000 K and predicted by DP model for the different amorphous systems.

Supplementary Note 2: Creating an amorphous structure

The amorphous structure was designed in 3 steps.

Step - 1: Firstly, a slab-like geometry of LiCl|GaF_3 was constructed, where a multilayer slab of [001] LiCl and [001] GaF_3 was vertically "|" interfaced and separated by 3 Å. A maximum strain of ~5 % was applied to match the lattice constants of LiCl and GaF_3 slabs in the LiCl|GaF_3 geometry and the molar ratio of LiCl:GaF_3 was 2. This structure had a total of 320 atoms and the lattice constant along the *z* direction was ~2.5 nm. Subsequently, to equilibrate the interface, the structure was subjected to AIMD simulation for t = 5 ps at T = 800 K, and then relaxed at T = 0 K to a local energy minimum. The obtained structure is shown in Supplementary Figure 6a.

Step - 2: The structure from the previous step as shown in Supplementary Figure 6a was then periodically repeated along the *x* and *y* directions (perpendicular to the interface) and an aperiodic cubic "*particle*" of size ~2.5 nm (Supplementary Figure 6b) was cut out from it. The center (middle point) of the aperiodic cubic particle was the same as the center of the structure in Supplementary Figure 6a. Eight such identical aperiodic *particles* were then arranged in a large supercell of size ~5.4 nm to obtain a structure containing 9088 (~10k) atoms (Supplementary Figure 6b). Each of the ~2.5 nm *particles* were rotated 90° with respect to their neighbors to ensure that the axis normal to the interface between LiCl and GaF_3 did not point in the same direction between neighboring *particles*. This rotation was performed to create a more randomized configuration of atoms. Domains of LiCl and GaF_3 in the initial large structure with ~10k atoms can be seen (Supplementary Figure 6b). The ratio of LiCl:GaF_3 was 2. The initial structure is representative of that in experiments, where during ball-milling, LiCl and GaF_3 particles are interfaced with each other and react.

Step - 3: Subsequently, the structure with 9088 (~10k) atoms (Supplementary Figure 6b) was relaxed in LAMMPS at T = 0 K to a local energy minimum, and then equilibrated in a high-temperature classical MD NVT simulation using LAMMPS for t = 50 ps at T = 900 K to allow mixing of the atoms. To obtain a representative structure in which domains of reactant materials are visible, an atomic configuration at t = 50 ps was chosen for further evaluation. This configuration was relaxed in LAMMPS at T = 0 K to a local energy minimum and then equilibrated (using LAMMPS) under the NPT ensemble for t = 1 ns at T = 300 K and zero external stress until there was no further change in density, lattice constant, angles, or total potential energy. The resulting amorphous structure shown in Supplementary Figure 6c, was then used in the subsequent analysis.

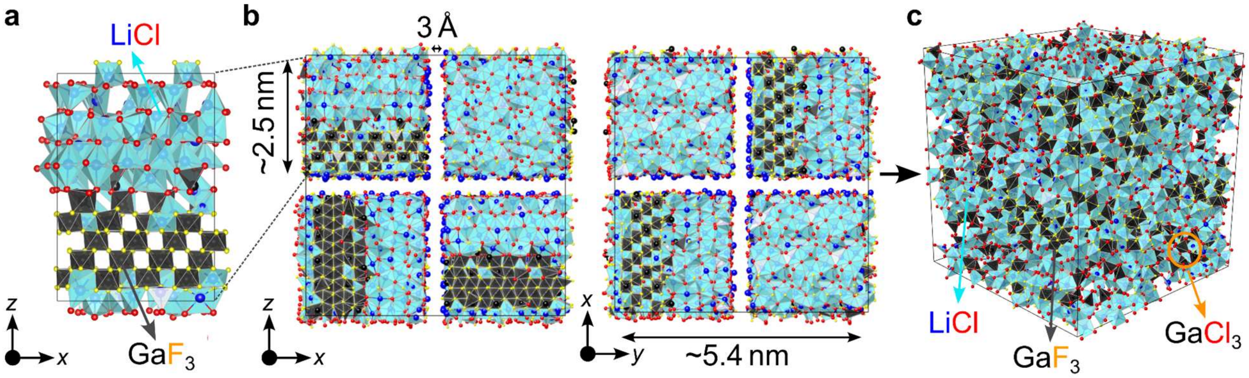

Supplementary Figure 6: (a) A slab-like geometry of LiCl|GaF_3, where a multilayer slab of [001] LiCl and [001] GaF_3 is "|" interfaced in a vertical geomtery. (b) Two different views of a large atomic structure of 2LiCl:GaF_3 containing 9088 (~10k) atoms and constructed from 8 identical aperiodic cubic *particles* of size ~2.5 nm. The *aperiodic* cubic particle was cut out from the *x*, *y* periodically repeated structure in (a). (c) An amorphous atomic structure representing 2LiCl:GaF_3 obtained after high temperature MD simulations of structure in (b) (details above in Supplementary Note 2)

Supplementary Note 2.1: Effect of heating temperature on the behavior of the amorphous system

In addition to the heating simulation at T = 900 K (Supplementary Figure 6), we also did similar simulations at T = 1200 K and T = 1500 K. Supplementary Figure 7*a* shows the element-wise radial pair distribution function g(r) before and after the high temperature (T = 900 K, 1200 K, and 1500 K) MD simulation of t = 50 ps. We find that in all the three temperature cases, the Ga-F and Li-Cl peak decrease, while the Ga-Cl and Li-F peak increase, indicating that anion exchange occurs when the two salts are mixed. This anion exchange is consistent with the thermodynamic driving force and with the experimental EXAFS (Figure 3a, main text). The Ga-F and Li-Cl peak decreases by larger amounts on increasing the temperature from 900 K - 1500 K, indicating that the extent of the anion exchange increases with increasing the temperature. However, anion exchange behavior is still captured by simulations at T = 900 K, which is consistent with experimental EXAFS (Figure 3a, main text). We also simulated the shear stress-strain response of the systems obtained after heating MD runs at three different temperatures. A procedure similar to that described in the main text for strain-controlled MD simulation was used to obtain the stress-strain response. Supplementary Figure 7b shows the shear stress-strain response of the structures at an applied shear strain rate of 10^9 s^{-1} on the *yz* plane. The stress-strain response curve of the 3 different structures looks very similar, indicating that they show similar mechanical behavior. We believe that T = 900 K is sufficient to capture the reaction mechanism and mechanical behavior of the amorphous system.

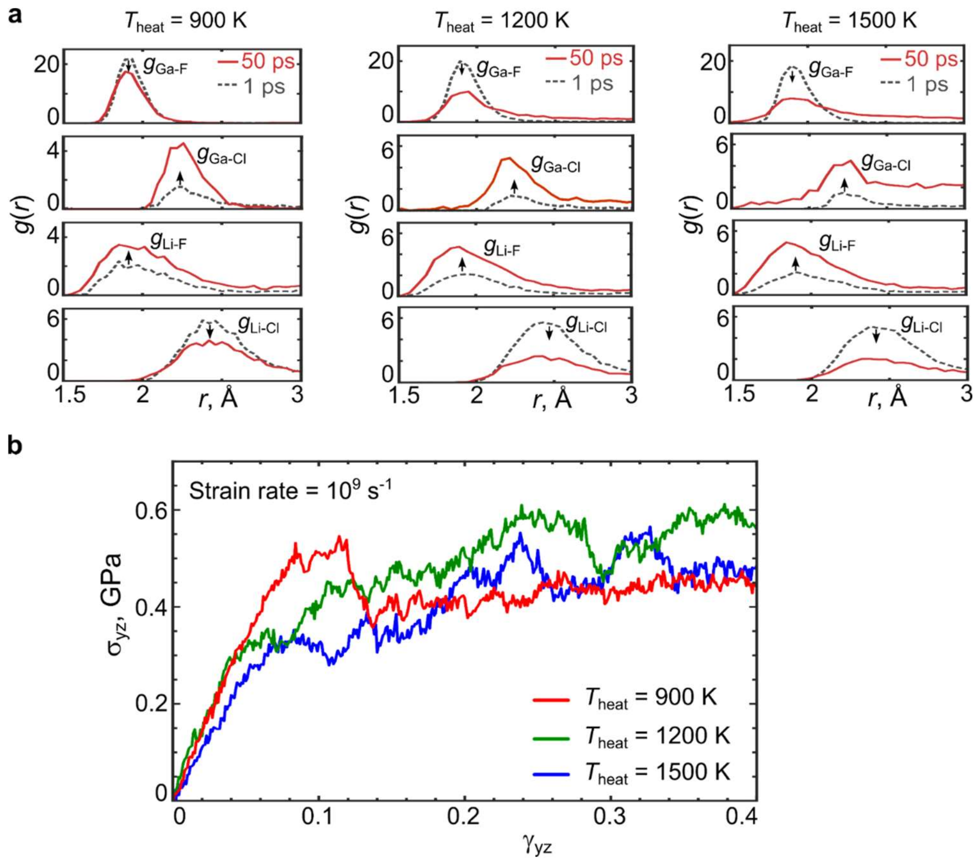

Supplementary Figure 7: (a) The element-wise radial pair distribution function $g(r)$ plotted at t = 1 ps and t = 50 ps of the MD simulation at T = 900 K, T = 1200 K, and T = 1500 K. (b) Shear stress (σ_{yz}) strain (γ_{yz}) response of the amorphous structures at T = 300 K at a constant applied shear strain rate of 10^9 s^{-1}. The three curves correspond to the behavior of the three systems obtained after heating MD runs at T = 900 K, T = 1200 K, and T = 1500 K.

Supplementary Note 3: Interface reaction LiCl+GaF_3: maximum thermodynamic driving force and most likely products

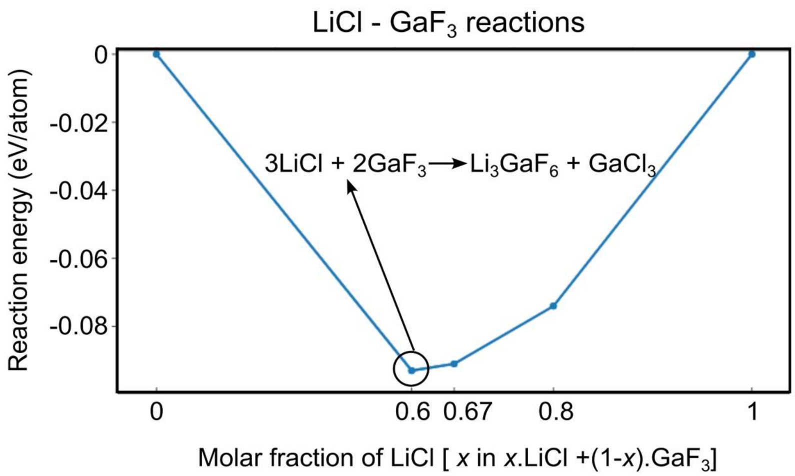

Supplementary Figure 8: The reaction energy of LiCl - GaF_3 reaction, taken from entries in the Materials Project database.

All salt precursors considered in this study for the possibility of soft-clay formation. Interface reaction among salt precursors with maximum thermodynamic driving force (most negative reaction energy E_{rxn}), taken from entries in the Materials Project database.

1. $3LiCl + 2GaF_3 \rightarrow 1Li_3GaF_6 + 1GaCl_3$, **$E_{rxn}$** = -93 meV/atom
2. $3LiI + 1GaF_3 \rightarrow 3LiF + 1GaI_3$, **$E_{rxn}$** = -188 meV/atom
3. $3LiCl + 1SbF_3 \rightarrow 3LiF + 1SbCl_3$, **$E_{rxn}$** = -74 meV/atom
4. $3LiI + 1InBr_3 \rightarrow 3LiBr + 1InI_3$, **$E_{rxn}$** = -65 meV/atom

Interface reaction with maximum thermodynamic driving force between salt precursors considered for soft-clay formation in a prior work[2]. The data is taken from entries in the Materials project database.

1. $0.412GaF_3 + 0.588NaCl \rightarrow 0.147NaGaCl_4 + 0.088Na_5Ga_3F_{14}$ **E_{rxn}** = -45 meV/atom
2. $0.857LiCl + 0.143InF_3 \rightarrow 0.143Li_3InCl_6 + 0.429LiF$ **E_{rxn}** = -68 meV/atom
3. $0.25LiCl + 0.75Ga_2O_3 \rightarrow 0.25LiGa_5O_8 + 0.25GaClO$ **E_{rxn}** = -1 meV/atom
4. $0.25GaF_3 + 0.75LiOH \rightarrow 0.25GaHO_2 + 0.25H_2O + 0.75LiF$ **E_{rxn}** = -197 meV/atom
5. $0.667Li_2O + 0.333GaF_3 \rightarrow 0.333LiGaO_2 + LiF$ **E_{rxn}** = -442 meV/atom
6. $3LiCl + 1LaF_3 \rightarrow$ No reaction
7. $0.4GaF_3 + 0.6LiBr \rightarrow 0.2\underline{GaBr_3} + 0.2Li_3GaF_6$ **E_{rxn}** = -128 meV/atom ($GaBr_3$ is a molecular solid)
8. $3NaCl + GaF_3 \rightarrow 3NaF + GaCl_3$ **E_{rxn}** = 0.474 eV (46 kJ mol^{-1})

Supplementary Table 3: Different possible molecular solid (MS) compounds and the corresponding mp-id obtained from the Materials Project (mp) database. The highlighted compounds are discussed in more detail in the main text. Possible anion exchange reaction with the most negative reaction energy E_{rxn} to form the MS is also listed. Li-based salts were considered as one of the reactants in the anion exchange reaction.

mp-id	MS Compounds	Reaction	E_{rxn} (eV/atom)
mp-29831	TaCl5	0.455 LiCl + 0.545 TaF5 → 0.091 TaCl5 + 0.455 LiTaF6	-0.097
mp-28391	SiCl4	0.571 LiCl + 0.429 SiF4 → 0.286 Li2SiF6 + 0.143 SiCl4	-0.036
mp-23250	UCl6	0.857 LiCl + 0.143 UF6 → 0.143 UCl6 + 0.857 LiF	-0.037
mp-22897	HgCl	0.5 LiCl + 0.5 HgF → 0.5 HgCl + 0.5 LiF	-0.23
mp-30086	GeCl4	0.429 GeF4 + 0.571 LiCl → 0.143 GeCl4 + 0.286 Li2GeF6	-0.173
mp-23280	AsCl3	0.75 LiCl + 0.25 AsF3 → 0.25 AsCl3 + 0.75 LiF	-0.098
mp-23290	PtCl2	0.667 LiCl + 0.333 PtF2 → 0.333 PtCl2 + 0.667 LiF	-0.362
mp-22908	BiCl3	0.75 LiCl + 0.25 BiF3 → 0.25 BiCl3 + 0.75 LiF	-0.063
mp-570355	PbCl4	0.8 LiCl + 0.2 PbF4 → 0.2 PbCl4 + 0.8 LiF	-0.213
mp-30952	**GaCl3**	0.6 LiCl + 0.4 GaF3 → 0.2 GaCl3 + 0.2 Li3GaF6	-0.093
mp-22855	HgCl2	0.667 LiCl + 0.333 HgF2 → 0.333 HgCl2 + 0.667 LiF	-0.289
mp-22872	**SbCl3**	0.75 LiCl + 0.25 SbF3 → 0.25 SbCl3 + 0.75 LiF	-0.074
mp-571518	WCl6	0.143 WF6 + 0.857 LiCl → 0.857 LiF + 0.143 WCl6	-0.122
mp-23176	SbCl5	0.455 LiCl + 0.545 SbF5 → 0.455 LiSbF6 + 0.091 SbCl5	-0.167
mp-23307	NbCl5	0.545 NbF5 + 0.455 LiCl → 0.455 LiNbF6 + 0.091 NbCl5	-0.088
mp-30092	TiCl4	0.571 LiCl + 0.429 TiF4 → 0.286 Li2TiF6 + 0.143 TiCl4	-0.068
mp-29866	SnCl4	0.571 LiCl + 0.429 SnF4 → 0.286 Li2SnF6 + 0.143 SnCl4	-0.171
mp-570005	SbBr3	0.75 LiBr + 0.25 SbF3 → 0.25 SbBr3 + 0.75 LiF	-0.126
mp-568846	TaBr5	0.455 LiBr + 0.545 TaF5 → 0.091 TaBr5 + 0.455 LiTaF6	-0.101
mp-570285	SiBr4	0.571 LiBr + 0.429 SiF4 → 0.286 Li2SiF6 + 0.143 SiBr4	-0.036
mp-1208424	TeBr4	0.8 LiBr + 0.2 TeF4 → 0.2 TeBr4 + 0.8 LiF	-0.181
mp-23317	AsBr3	0.75 LiBr + 0.25 AsF3 → 0.25 AsBr3 + 0.75 LiF	-0.152
mp-23177	HgBr	0.5 LiBr + 0.5 HgF → 0.5 HgBr + 0.5 LiF	-0.301
mp-1207486	ZrBr4	0.571 LiBr + 0.429 ZrF4 → 0.286 Li2ZrF6 + 0.143 ZrBr4	-0.021
mp-27642	PaBr5	0.833 LiBr + 0.167 PaF5 → 0.167 PaBr5 + 0.833 LiF	-1.54
mp-23292	HgBr2	0.667 LiBr + 0.333 HgF2 → 0.333 HgBr2 + 0.667 LiF	-0.385
mp-27399	SbBr3	0.75 LiBr + 0.25 SbF3 → 0.25 SbBr3 + 0.75 LiF	-0.126
mp-28601	NbBr5	0.545 NbF5 + 0.455 LiBr → 0.455 LiNbF6 + 0.091 NbBr5	-0.098
mp-30953	**GaBr3**	0.4 GaF3 + 0.6 LiBr → 0.2 GaBr3 + 0.2 Li3GaF6	-0.128
mp-569814	TiBr4	0.571 LiBr + 0.429 TiF4 → 0.286 Li2TiF6 + 0.143 TiBr4	-0.081
mp-574086	SiBr4	0.571 LiBr + 0.429 SiF4 → 0.286 Li2SiF6 + 0.143 SiBr4	-0.036
mp-23216	SnBr4	0.429 SnF4 + 0.571 LiBr → 0.286 Li2SnF6 + 0.143 SnBr4	-0.209
mp-23288	AlBr3	no reaction	
mp-567604	GeBr4	0.429 GeF4 + 0.571 LiBr → 0.143 GeBr4 + 0.286 Li2GeF6	-0.2
mp-1203015	TaBr5	0.455 LiBr + 0.545 TaF5 → 0.091 TaBr5 + 0.455 LiTaF6	-0.101
mp-30954	**GaI3**	0.25 GaF3 + 0.75 LiI → 0.25 GaI3 + 0.75 LiF	-0.188
mp-1078195	SiI3	0.5 LiI + 0.5 SiF3 → 0.167 SiI3 + 0.25 Li2SiF6 + 0.083 Si	-0.056
mp-23218	AsI3	0.75 LiI + 0.25 AsF3 → 0.25 AsI3 + 0.75 LiF	-0.248
mp-567789	**InI3**	0.75 LiI + 0.25 InF3 → 0.25 InI3 + 0.75 LiF	-0.225
mp-22859	HgI	0.5 LiI + 0.5 HgF → 0.5 HgI + 0.5 LiF	-0.385
mp-23182	SnI4	0.2 SnF4 + 0.8 LiI → 0.2 SnI4 + 0.8 LiF	-0.291
mp-570884	TeI4	0.8 LiI + 0.2 TeF4 → 0.2 TeI4 + 0.8 LiF	-0.287
mp-29109	SiI3	no reaction	
mp-30930	AlI3	no reaction	
mp-635441	SiI4	0.571 LiI + 0.429 SiF4 → 0.286 Li2SiF6 + 0.143 SiI4	-0.054
mp-23266	GeI4	0.2 GeF4 + 0.8 LiI → 0.2 GeI4 + 0.8 LiF	-0.257

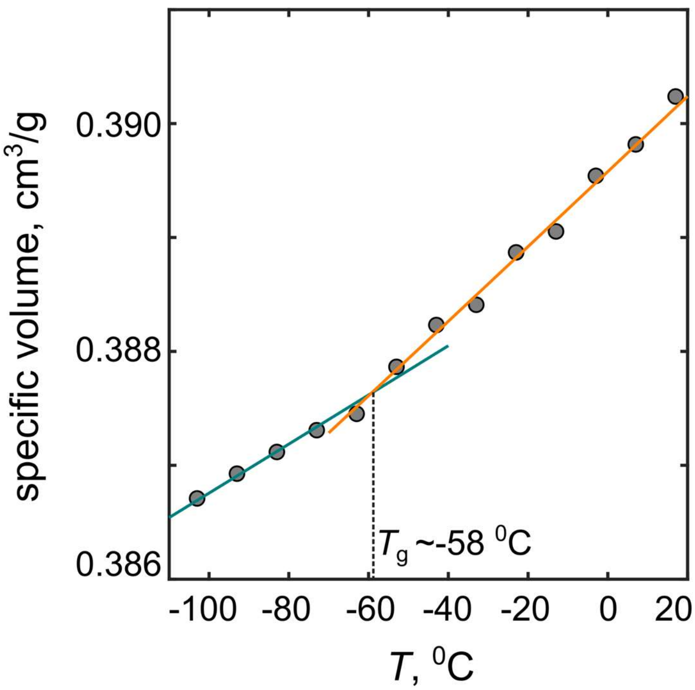

Supplementary Figure 9: The specific volume vs temperature plot obtained from the MD simulations of amorphous 2LiCl-1GaF$_3$, illustrating a glass-like phase transition at T_g ~ -58 ^{o}C. The structures were equilibrated in LAMMPS for t = 2 ns at different temperatures under the NPT ensemble.

Supplementary Note 4: Experimental results: XRD spectra and EIS

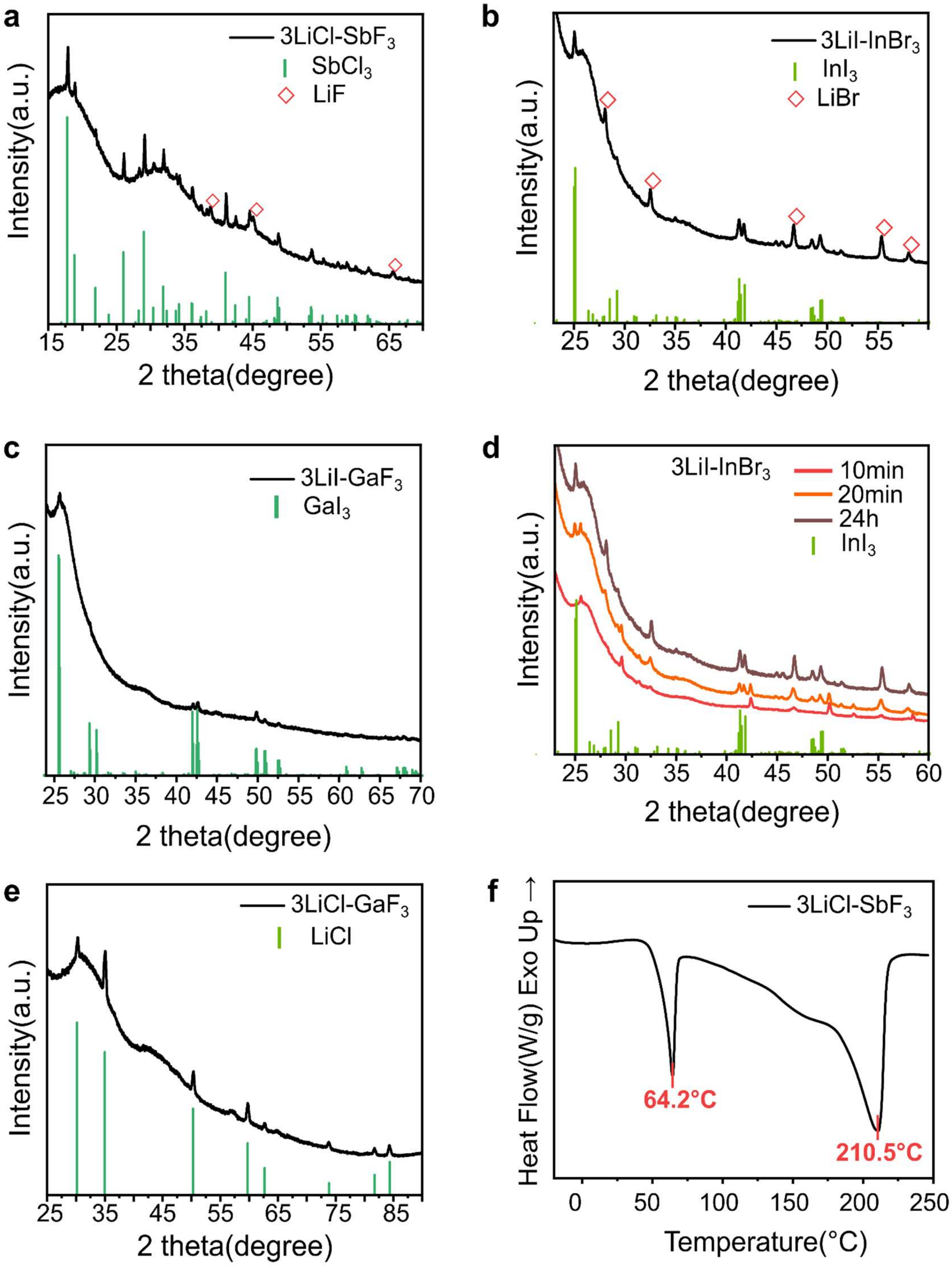

Supplementary Figure 10: (a), (b), (c), (e) X-ray diffraction (XRD) spectra of different materials after ball milling (BM) and the peak assignments of the products formed. (d) XRD spectra of 3LiI-$InBr_3$ with time during BM. The InI_3 peak appears just after 20 mins of BM. (f) Differential scanning calorimetry (DSC) analysis of 3LiCl-1SbF_3 material after BM. An endothermic peak around ~64 ^{o}C and ~210 ^{o}C corresponds to melting and boiling of $SbCl_3$, which is formed during BM.

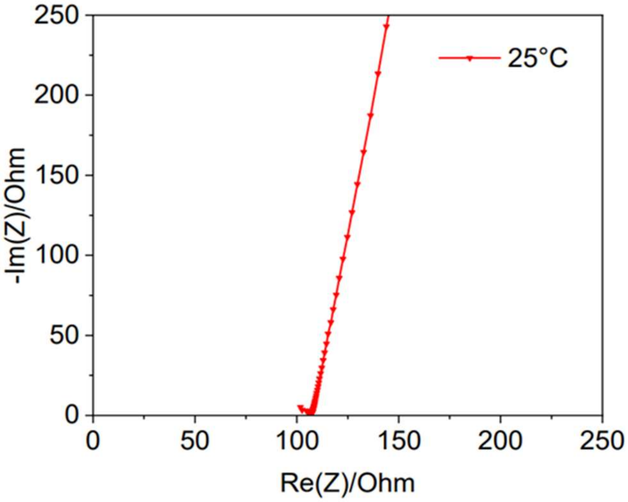

Supplementary Figure 11: Electrochemical Impedance Spectroscopy (EIS) measurements showing the Nyquist plot used to determine the Li-ion conductivity in 2LiCl-1GaF_3 soft clay-like material.

Supplementary Note 5: Details of non-affine displacement (D^2_{min}) calculations

A similar procedure as outlined before[3] was used to calculate the non-affine displacement D^2_{min} (Supplementary Eq. 1-4).

$$D^2_{min}(t,\Delta t) = \Sigma_n \Sigma_i (r^i_n(t) - r^i_0(t) - \Sigma_j(\delta_{ij} + \varepsilon_{ij}) \times [r^j_n(t-\Delta t) - r^j_0(t-\Delta t)])^2 \quad \text{Supplementary Eq. 1}$$

$$\varepsilon_{ij} = \Sigma_k X_{ik} Y^{-1}_{ik} - \delta_{ij} \quad \text{Supplementary Eq. 2}$$

$$X_{ij} = \Sigma_n [r^i_n(t) - r^i_0(t)] \times [r^j_n(t-\Delta t) - r^j_0(t-\Delta t)] \quad \text{Supplementary Eq. 3}$$

$$Y_{ij} = \Sigma_n [r^i_n(t-\Delta t) - r^i_0(t-\Delta t)] \times [r^j_n(t-\Delta t) - r^j_0(t-\Delta t)] \quad \text{Supplementary Eq. 4}$$

where the indices *i* and *j* denote spatial coordinates and the index *n* runs over the atoms within the interaction range (r_{cut}) of the reference atom n_0. Here, r_{cut} = 4 Å was used. $r_n^i(t)$ is the ith component of the position of the nth atom at time *t*. $D^2_{min}(t, \Delta t)$ is then the local deviation from affine deformation during the time interval $t - \Delta t$, *t*. This is implemented in the Ovito software.

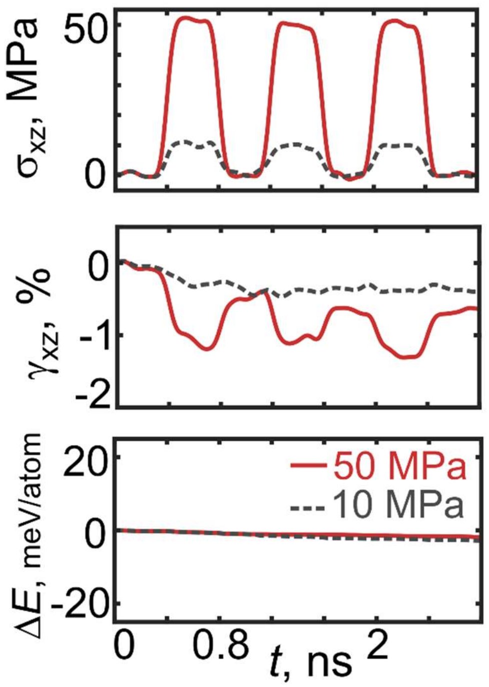

Supplementary Figure 12: The applied external shear stress σ_{xz}, accumulated shear strain γ_{xz}, and total potential energy change ΔE of the amorphous structure as a function of time in the MD simulation at T = 300 K. The external shear stress ranged from 10 to 50 MPa. The accumulated shear strain after three cycles is non-negligible ($\gamma_{xz} \neq 0$), signifying permanent deformation. Here, the *xz*-component of external stress was applied and other components were kept at zero (using a similar procedure as used for Figure 1c (main text)).

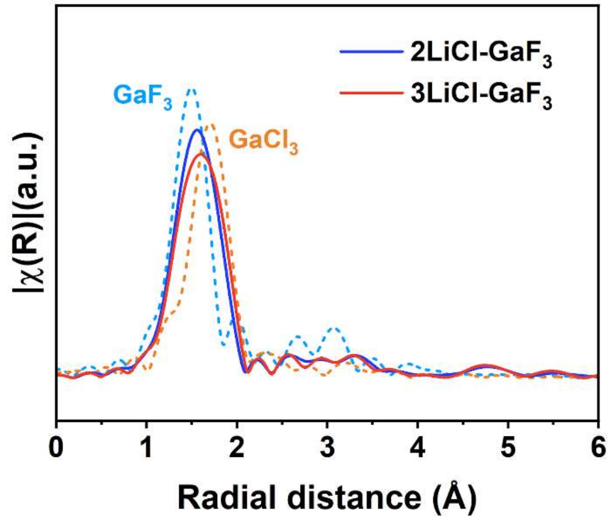

Supplementary Figure 13: Ga K-edge EXAFS of different materials, bulk GaF_3, bulk $GaCl_3$, and clay-like 2LiCl-1GaF_3 and 3LiCl-1GaF_3. $|\chi(r)|$, magnitude of Fourier transformed EXAFS.

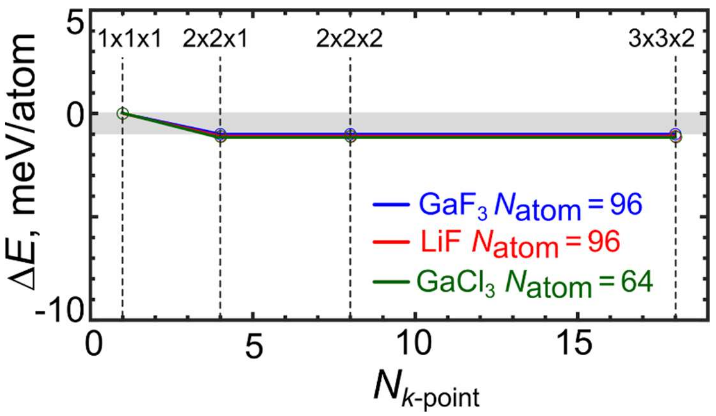

Supplementary Figure 14: The energy of the different chemical systems obtained using DFT calculations with different *k*-grid sizes. $N_{k\text{-point}}$ is the total number of *k*-points in the *k*-grid. The energy is referenced to the value obtained with one *k*-point.

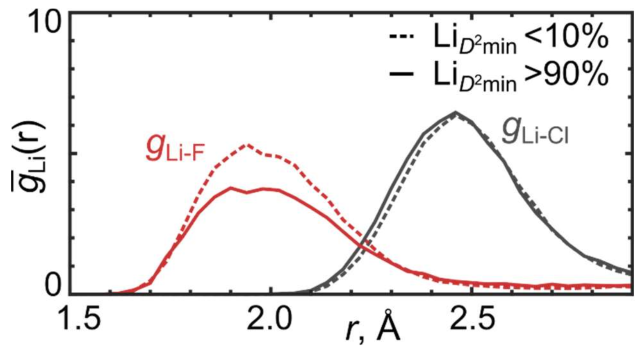

Supplementary Figure 15: The Li-Cl and Li-F pair distribution function *g*(r) of Li-atoms with largest (D^2_{min} > 90% of maximum) and smallest (D^2_{min} < 10% of maximum) non-affine displacement values. The *g*(*r*) values were averaged over the strain interval γ_{yz} = 0.09 - 0.14. The Li atoms in the areas involved in plastic deformation have a Cl-rich environment, as evidenced by the lower Li-F peak for Li atoms with D^2_{min} >90% of maximum compared to those with D^2_{min} values <10% of maximum.

Supplementary References

1. He, X., Zhu, Y., Epstein, A. & Mo, Y. Statistical variances of diffusional properties from ab initio molecular dynamics simulations. *Npj Comput. Mater.* **4**, 1–9 (2018).
2. Jung, S.-K. *et al.* Pliable Lithium Superionic Conductor for All-Solid-State Batteries. *ACS Energy Lett.* **6**, 2006–2015 (2021).
3. Falk, M. L. & Langer, J. S. Dynamics of viscoplastic deformation in amorphous solids. *Phys. Rev. E* **57**, 7192–7205 (1998).
